# Hyperglycemia Induces Inflammatory Response of Human Macrophages to CD163-Mediated Scavenging of Hemoglobin-Haptoglobin Complexes

**DOI:** 10.3390/ijms23031385

**Published:** 2022-01-26

**Authors:** Laura Matuschik, Vladimir Riabov, Christina Schmuttermaier, Tatyana Sevastyanova, Christel Weiss, Harald Klüter, Julia Kzhyshkowska

**Affiliations:** 1 Institute of Transfusion Medicine and Immunology, Mannheim Institute for Innate Immunoscience (MI3), Medical Faculty Mannheim, Heidelberg University, 68167 Mannheim, Germany; Vladimir.Ryabov@medma.uni-heidelberg.de (V.R.); Christina.Schmuttermaier@medma.uni-heidelberg.de (C.S.); Tatyana.Sevastyanova@medma.uni-heidelberg.de (T.S.); harald.klueter@medma.uni-heidelberg.de (H.K.); 2 Center of Surgery, Department of General and Visceral Surgery, Medical Center—University of Freiburg, Faculty of Medicine, 79106 Freiburg, Germany; 3 Department of Medical Statistics and Biomathematics, Medical Faculty Mannheim, University of Heidelberg, 68167 Mannheim, Germany; Christel.Weiss@medma.uni-heidelberg.de; 4 German Red Cross Blood Service Baden-Württemberg-Hessen, 68167 Mannheim, Germany

**Keywords:** diabetes mellitus, hyperglycemia, macrophages, inflammation, CD163, scavenger receptor, hemoglobin-haptoglobin complexes

## Abstract

Hyperglycemia, a hallmark of diabetes, can induce inflammatory programming of macrophages. The macrophage scavenger receptor CD163 internalizes and degrades hemoglobin-haptoglobin (Hb-Hp) complexes built due to intravascular hemolysis. Clinical studies have demonstrated a correlation between impaired scavenging of Hb-Hp complexes via CD163 and diabetic vascular complications. Our aim was to identify whether hyperglycemia is able to amplify inflammation via Hb-Hp complex interactions with the immune system. M(IFNγ), M(IL-4), and control M0 macrophages were differentiated out of primary human monocytes in normo- (5 mM) and hyperglycemic (25 mM) conditions. CD163 gene expression was decreased 5.53 times in M(IFNγ) with a further decrease of 1.99 times in hyperglycemia. Hyperglycemia suppressed CD163 surface expression in M(IFNγ) (1.43 times). Flow cytometry demonstrated no impairment of Hb-Hp uptake in hyperglycemia. However, hyperglycemia induced an inflammatory response of M(IFNγ) to Hb-Hp1-1 and Hb-Hp2-2 uptake with different dynamics. Hb-Hp1-1 uptake stimulated IL-6 release (3.03 times) after 6 h but suppressed secretion (5.78 times) after 24 h. Contrarily, Hb-Hp2-2 uptake did not affect IL-6 release after 6h but increased secretion after 24 h (3.06 times). Our data show that hyperglycemia induces an inflammatory response of innate immune cells to Hb-Hp1-1 and Hb-Hp2-2 uptake, converting the silent Hb-Hp complex clearance that prevents vascular damage into an inflammatory process, hereby increasing the susceptibility of diabetic patients to vascular complications.

## 1. Introduction

Diabetes mellitus is a heterogeneous group of metabolic disorders sharing the common ground of chronic hyperglycemia that induces micro- and macrovascular complications [1,2]. Large clinical trials, i.e., the Diabetes Control and Complications Trial (DCCT) and the United Kingdom Prospective Diabetes Study (UKPDS), found that the duration and degree of hyperglycemia correlates with the extent of microvascular complications [3,4]. Hyperglycemia can cause vascular complications by direct and indirect mechanisms, by the activation of detrimental inflammatory pathways in endothelial cells or by activation of immune cells, primarily of myeloid origin [5,6,7,8]. It was suggested that subclinical chronic systemic inflammation creates conditions for vascular damage [9,10,11]. Chronic inflammation is characterized by only moderate elevation of inflammatory cytokines, e.g., TNFα, IL-1β, and IL-6, induced by exogenous or endogenous factors [12]. The major source of inflammatory cytokines in chronic inflammation are macrophages. We have demonstrated previously that human macrophages respond to hyperglycemia by elevated production of predominantly proinflammatory cytokines [6,13]. Hyperglycemia can induce an inflammatory program in innate immune cells on the epigenetic level, as we showed for the enhanced presence of activating histone marks on the promoters of *S100A9* and *S100A12* genes, responsible for vascular inflammation in diabetes [7,14,15,16]. However, macrophages control inflammation not only by the release of proinflammatory factors, but also by their complex scavenging activity, which can be silent and tolerogenic, or can provoke additional inflammation. The effect of hyperglycemia on this essential function of monocytes and macrophages is unexplored.

CD163 is a scavenger receptor expressed on circulating monocytes and on tissue macrophages in different pathologies [17,18,19,20]. CD163 controls inflammation by the internalization and degradation of hemoglobin-haptoglobin (Hb-Hp) complexes [21]. Expression of CD163 is controlled by pro- and anti-inflammatory factors, where anti-inflammatory agents, such as glucocorticoids and IL-10, stimulate CD163 expression, and proinflammatory cytokines, such as IFNγ and TNFα, suppress CD163 expression [22,23,24,25,26]. The anti-inflammatory cytokine IL-4 had no effect on CD163 protein expression [23,27]. 

The most comprehensively characterized function of CD163 is its homeostatic role in the scavenging of Hb-Hp complexes formed as a result of intravascular hemolysis. This mechanism physiologically occurs for 10–20% of erythrocytes and increases considerably in pathological conditions, e.g., hemoglobinopathies or inflammation [21,28]. The formation of Hb-Hp complexes protects the endothelium and kidneys from the toxicity of free hemoglobin by preventing renal deposition of free hemoglobin resulting in parenchymal and vascular damage [28,29]. 

Hb-Hp complexes bind with high affinity to membrane-bound CD163 of both circulating monocytes and tissue macrophages, leading to their internalization and degradation via the cytoprotective and anti-inflammatory heme oxygenase-1 [21,30,31]. This anti-inflammatory capacity is decreased in diabetes mellitus, as *CD163* mRNA expression in peripheral blood mononuclear cells (PBMCs) is significantly suppressed in newly diagnosed diabetic patients [32]. Moreover, the percentage of macrophages in atherosclerotic plaques and PBMC expressing CD163 was significantly reduced in diabetic patients compared to non-diabetic individuals [28,33]. Haptoglobin, the protein binding to free extracorpuscular hemoglobin, exists in two known allelic variants: *Hp1* and *Hp2*, leading to three possible phenotypes: Hp1-1, Hp1-2, and Hp2-2 [34]. Haptoglobin serum levels differ considerably from 0.3–3.0 mg/mL between healthy individuals but stay reasonably constant for one individual, and are saturated when 500–1500 mg/L free hemoglobin is present in serum [34,35]. During inflammation, haptoglobin, which is a hepatocyte-produced acute-phase protein, is induced 2–5-fold by the acute-phase mediators IL-1 and IL-6 and can be released locally from storage granules by active neutrophils [36,37]. The clearance and antioxidant capacity of individuals expressing Hp1-1 has been observed to be superior to in Hp2-2 individuals [38,39]. Longitudinal prospective studies have demonstrated that the Hp2-2 phenotype is an independent risk factor for the development of cardiovascular disease and increased susceptibility to vascular complications in diabetic individuals in comparison with the homozygous Hp1-1 variant [33,34,40]. As a possible mechanism for the proneness to vascular complications, it has been demonstrated that CD163 is downregulated in macrophages in atherosclerotic plaques of diabetic patients with the *Hp2-2* genotype, indicating a compromised hemoglobin clearing capability [33]. 

Although clinical studies found a correlation between the impaired scavenging of Hb-Hp complexes in diabetes mellitus, the effect of hyperglycemia on the expression and scavenging activity of CD163 in primary human macrophages has not been studied to date. 

We hypothesized that hyperglycemia might have a novel mechanism of amplifying inflammation via Hb-Hp complex interactions with the immune system, as hyperglycemia has already been identified as a direct driver of inflammatory cytokine release in monocytes and macrophages. In this study, we focused on the essential monocyte/macrophage clearance functions required to eliminate Hb-Hp complexes. We specifically analyzed the differences between Hp1-1 and Hp2-2 effects in hyperglycemic conditions in terms of the dynamics and spectrum of this inflammatory response relevant to the clinically observed susceptibility of diabetic patients expressing the Hp2-2 variant to the development of vascular complications.

## 2. Results

### 2.1. IFNγ and IL-4 Suppress CD163 Expression in Macrophages, and Hyperglycemia Enhances the IFNγ Effect

**Figure 1:**
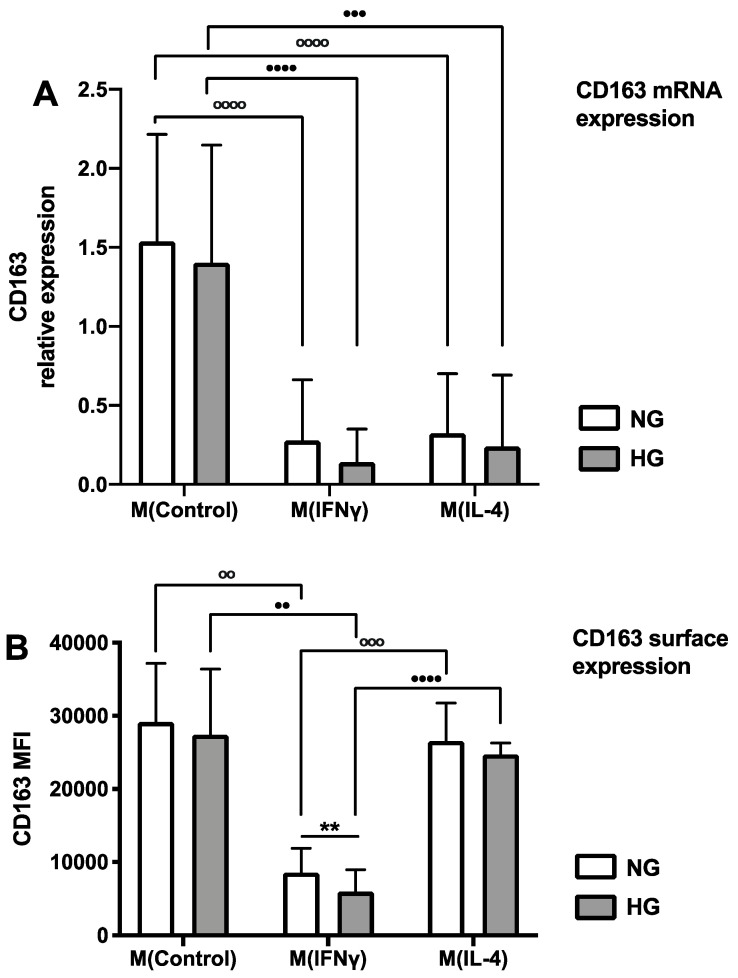
RT-PCR and flow cytometry analysis of CD163 expression in differentially stimulated macrophages. Monocyte-derived macrophages were cultured for 6 days in normal-glucose (5 mM, NG) and high-glucose (25 mM, HG) conditions and stimulated with either IFNγ or IL-4 or without further cytokines (M(Control)). (**A**): mRNA expression was measured by RT-PCR. Graph depicts the mean values of 8 donors ± SD. For statistical analysis, the Wilcoxon matched-pairs rank test was used. (**B**): Fluorescence intensity was measured by flow cytometry. Graph depicts the mean values of 6 donors ± SD. For statistical analysis, a paired *t* test was used. * denotes statistical significance between normal- and high-glucose conditions (** *p* < 0.01). ° denotes statistical significance between different cytokine stimulations in NG conditions (°° *p* < 0.01, °°° *p* < 0.001, °°°° *p* < 0.0001). • denotes statistical significance between different cytokine stimulations in HG conditions (•• *p* < 0.01, ••• *p* < 0.001, •••• *p* < 0.0001).

Human primary monocyte-derived macrophages were differentiated into homeostatic (M(Control)) M0 macrophages, inflammatory (M(IFNγ)), and healing (M(IL-4)) macrophages in normal and hyperglycemic conditions, and the gene expression of *CD163* was quantified by RT-PCR (Figure 1A). Hyperglycemia did not affect *CD163* expression in M0. IFNγ had a statistically significant inhibitory effect on *CD163* expression compared to M0 macrophages (5.53 times decrease, *p*-value < 0.0001) (Figure 1A). Hyperglycemia enhanced this effect 1.99 times; however, statistical significance was not reached due to individual donor responses (Figure 1A, individual donors shown in Supplementary Figure S4). The suppressing effect of IL-4 on *CD163* gene expression was very strong (fold change 4.76, *p*-value < 0.0001) but did not reach the suppressive level of IFNγ (Figure 1A). Hyperglycemia had almost no effect on the IL-4-mediated suppression of *CD163* gene expression. Flow cytometry quantification of CD163 surface expression demonstrated, similarly to gene expression, a strong suppressive effect of IFNγ compared to the control conditions (3.4 times, *p*-value < 0.01) (Figure 1B). Hyperglycemia had an additional statistically significant effect on the suppression of CD163 surface expression in M(IFNγ) (fold change 1.43, *p*-value < 0.01) (Figure 1B, data for individual donors are shown in Supplementary Figure S5). However, in contrast to gene expression levels, in M(IL-4), surface expression levels of CD163 did not change compared to M(Control), suggesting that an additional mechanism in healing macrophages can support sufficient levels of surface CD163 to ensure control of inflammatory responses. 

### 2.2. CD163-Mediated Internalization of Hb-Hp Complexes Is Efficient in NG and HG Conditions in M0 and M(IL-4) Macrophages

**Figure 2:**
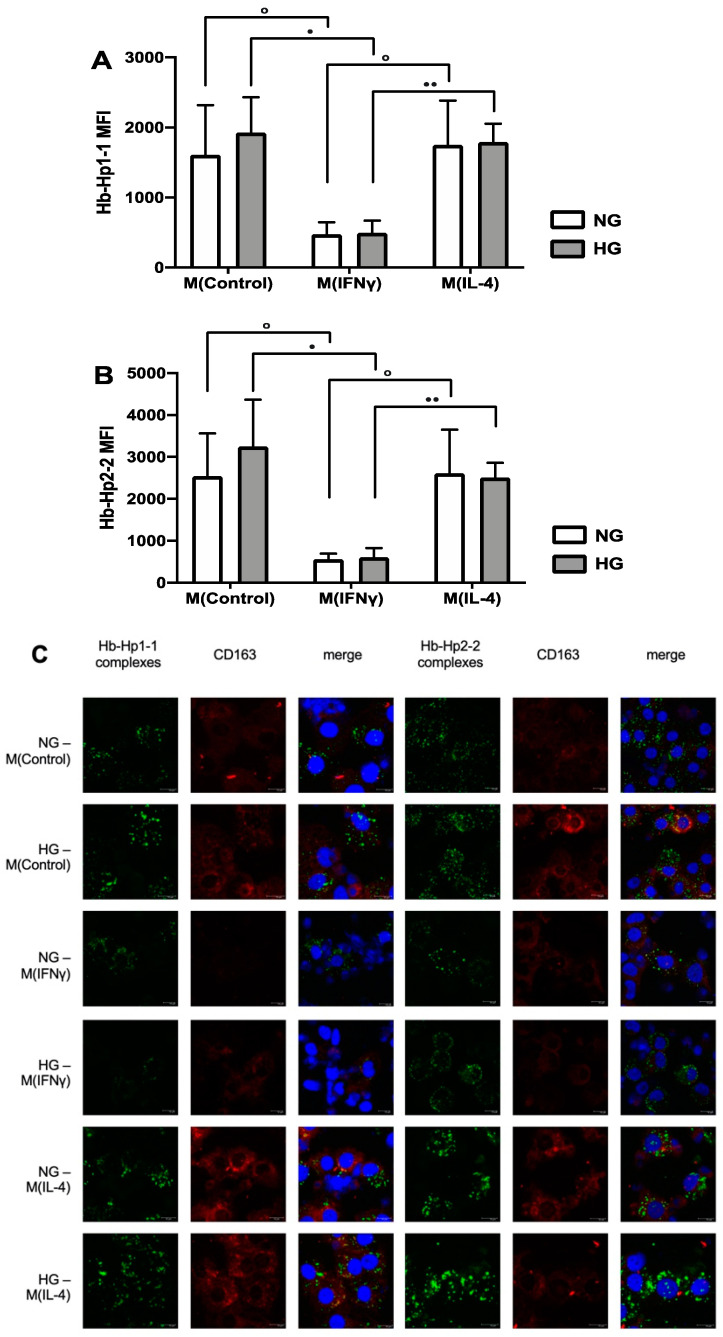
Endocytosis of Alexa Fluor^®^ 488-labeled Hb-Hp1-1-/Hp2-2-complexes in normoglycemic and hyperglycemic conditions in differentially activated macrophages. Endocytosis was performed on day 6 of macrophage differentiation (control, IFNγ-stimulated, and IL-4-stimulated) in normal (5 mM, NG) and high (25 mM, HG) glucose conditions. Macrophages were incubated with Hb-Hp1-1 or Hb-Hp2-2 complexes for 30 min. (**A**): flow cytometry analysis after stimulation with Hb-Hp1-1 complexes; (**B**): flow cytometry analysis after stimulation with Hb-Hp2-2 complexes. Mean fluorescence intensity (MFI) values shown on the graphs are after subtraction of ‘no ligand’ controls. Graphs depict the mean fluorescence intensity values of 5 individual donors ± SD. For statistical analysis, a paired *t* test was used. ° denotes statistical significance between different cytokine stimulations in NG conditions (° *p* < 0.05). • denotes statistical significance between different cytokine stimulations in HG conditions (• *p* < 0.05, •• *p* < 0.01). (**C**): Confocal microscopy analysis. Alexa Fluor^®^ 488-labeled Hb-Hp1-1-/Hp2-2-complexes are visualized in green. CD163 was detected with a goat polyclonal anti-human CD163 antibody and Cy3-labeled donkey anti-goat secondary antibody (shown in red). Nuclei were stained with DRAQ5 (shown in blue). Yellow-orange color indicates co-localization of Hb-Hp complexes and CD163. The scale bar indicates 10 µm.

To identify the functional consequences of the observed regulation of CD163, we examined the effect of hyperglycemia on the scavenging function of CD163. Therefore, we quantitatively evaluated the uptake of fluorescently labeled hemoglobin-haptoglobin complexes (Hb-Hp1-1 complexes and Hb-Hp2-2 complexes) by differentially stimulated macrophages via flow cytometry. M0 and M(IL-4) efficiently internalized both Hb-Hp1-1 (M(IL-4) compared to M(IFNγ) (fold change 3.7, *p*-value < 0.01, Figure 2A) and Hb-Hp2-2 complexes (M(IL-4) compared to M(IFNγ) (fold change 4.7, *p*-value < 0.01, Figure 2B) in normoglycemia. In hyperglycemic conditions, too, uptake of Hb-Hp1-1 and Hb-Hp2-2 was significantly impaired in M(IFNγ) compared to M0 and M(IL-4) (Hb-Hp1-1: M(IL-4) compared to M(IFNγ) (fold change 3.6, *p*-value < 0.001, Figure 2A) and Hb-Hp2-2: M(IL-4) compared to M(IFNγ) (fold change 4.2, *p*-value < 0.001, Figure 2B), which correlated with significantly suppressed surface levels of CD163 in M(IFNγ) (Figure 1B). There was no difference in the endocytosis efficiency of Hb-Hp1-1 and Hb-Hp2-2 in hyperglycemic conditions.

The internalization of Hb-Hp1-1 and Hb-Hp2-2 complexes was visualized by confocal microscopy and confirmed efficient endocytosis of complexes in M0 and M(IL-4), and only residual endocytosis in M(IFNγ) (Figure 2C). The endocytosis of Hb-Hp1-1 and Hb-Hp2-2 in hyperglycemic conditions was as efficient as in normoglycemic conditions. Even though the haptoglobin variants Hp1-1 and Hp2-2 differ considerably in their molecular structure [34], we observed the same uptake pattern for both complexes. The uptake efficiency was dependent on macrophage polarization, which defines the levels of CD163 surface exposure. There was no functional impairment of endocytosis of Hb-Hp1-1 and Hb-Hp2-2 via CD163 in hyperglycemia.

### 2.3. Hyperglycemia Enhances the Release of Inflammatory Cytokines in Response to Scavenging of Hemoglobin-Haptoglobin Complexes via CD163 in M(IFNγ)

**Figure 3:**
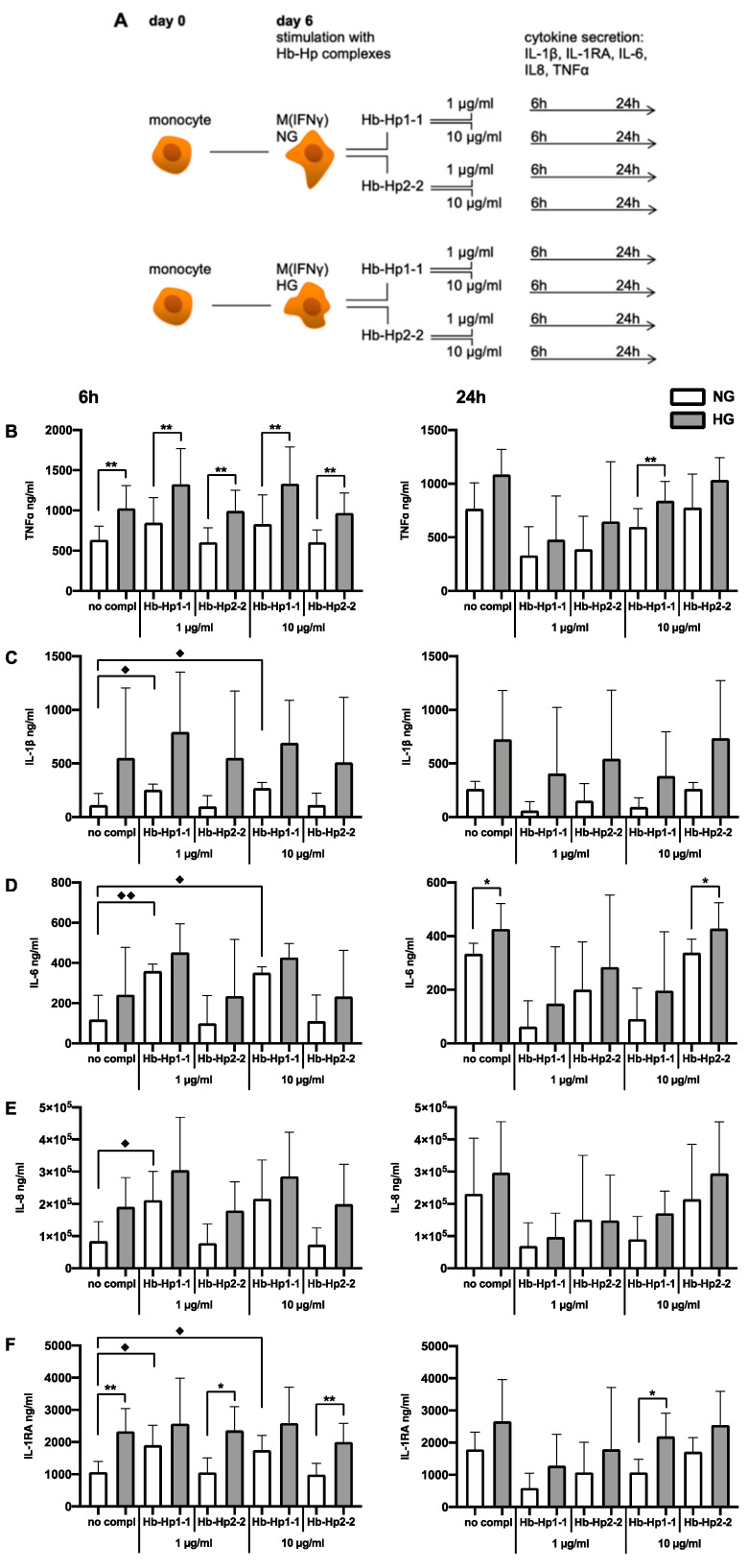
Effect of Hb-Hp complexes on the secretion of TNFα, IL-1β, IL-6, IL-8, and IL-1RA by IFNγ-stimulated human primary macrophages. Monocyte-derived macrophages were stimulated with IFNγ and cultured for 6 days in normal (5 mM, NG) and high (25 mM, HG) glucose conditions. On day 6, Hb-Hp1-1 and Hb-Hp2-2 complexes in 2 different concentrations (1 and 10 µg/mL) were added to the cells or, serving as a negative control, no complexes (‘no compl’) were added. The amounts of TNFα, IL-1β, IL-6, and IL-8 and IL-1RA were detected in the supernatants by the according ELISAs 6 and 24 h after stimulation with Hb-Hp complexes. (**A**): Schematic presentation of the experimental design used to examine whether stimulation with Hb-Hp complexes affects the inflammatory activation of M(IFNγ). (**B**): TNFα secretion; (**C**): IL-1β secretion; (**D**): IL-6 secretion; (**E**): IL-8 secretion; (**F**): IL-1RA secretion (mean values of 5 donors ± SD). For statistical analysis, paired *t* tests were used. * denotes statistical significance between normal- and high-glucose conditions (* *p* < 0.05, ** *p* < 0.01) ◆ denotes statistical significance between no stimulation and stimulation with Hb-Hp1-1 complexes after 6 h (◆ *p* < 0.05, ◆◆ *p* < 0.01).

Next, we analyzed whether the uptake of Hb-Hp complexes can affect inflammatory activation of macrophages. CD14^+^ monocytes of healthy donors were differentiated for 6 days in a serum-free medium complemented with IFNγ in normal (5 mM, NG) and high (25 mM, HG) glucose conditions. On day 6, macrophages were stimulated with Hb-Hp1-1 complexes or Hb-Hp2-2 complexes at concentrations of 1 and 10 µg/mL, as the haptoglobin level varies individually from 0.3-3 mg/mL and, as an acute-phase protein, its physiological plasma levels increase 2-5-fold in inflammation [34,36,37,41]. We selected a set of inflammatory cytokines (TNFα, IL-1β, IL-6, IL-8, and IL-1 receptor antagonist (IL-1RA)) as a read-out. The concentration of these cytokines was identified 6 and 24h after stimulation with Hb-Hp1-1 or Hb-Hp2-2 complexes (experimental design: Figure 3A). 

The secretion of our read-out cytokines tended to be enhanced in hyperglycemic conditions in the absence and also in the presence (after 6 and 24h) of Hb-Hp complex stimulation (Figure 3B–F).

The release of the acute-phase inflammatory cytokine TNFα was stimulated by Hb-Hp1-1 complexes after 6 h in NG, with this effect being significantly enhanced by hyperglycemia (Figure 3B). The increase in hyperglycemic conditions was similar for Hb-Hp1-1 complex concentrations of 1 (1.57 times, *p*-value < 0.01) and 10 µg/mL (1.61 times, *p*-value < 0.01). However, 24 h after the stimulation with Hb-Hp1-1 complexes, suppression of TNFα secretion was detected in both normo- and hyperglycemic conditions, with a statistically significant effect in hyperglycemia 24 h after stimulation with 10 µg/mL Hb-Hp1-1 complexes (1.29 times suppression, *p*-value < 0.05). The stimulation with Hb-Hp2-2 complexes per se had no statistically significant effect on TNFα release compared to non-stimulated macrophages (Figure 3B). However, hyperglycemia significantly enhanced TNFα release 6 h after stimulation with Hb-Hp2-2 complexes (1 µg/mL: 1.65 times, *p*-value < 0.01; 10 µg/mL: 1.61 times, *p*-value < 0.01) (Figure 3B). A statistically significant increase in TNFα secretion could only be detected 6h after the stimulation with Hb-Hp complexes in hyperglycemia, regardless of the present haptoglobin variant.

The release of proinflammatory IL-1β was significantly increased 6 h after the stimulation with Hb-Hp1-1 complexes in NG for both complex concentrations (1 µg/mL: 2.29 times, *p*-value < 0.05; 10 µg/mL: 2.43 times, *p*-value < 0.05, Figure 3C). Hyperglycemia elicited a strong enhancement of IL-1β release after stimulation with Hb-Hp complexes regardless of the Hp variant, with the strongest effect being detected 24 h after stimulation with 1 µg/mL Hb-Hp1-1 complexes (6.75 times). However, statistical significance was not reached due to individual donor responses. Then, 24 h after the stimulation with Hb-Hp1-1 complexes, IL-1β secretion was suppressed in both normo- and hyperglycemic conditions, with the strongest suppression detected in NG after stimulation with 1 µg/mL Hb-Hp1-1 complexes (4.21 times, *p*-value < 0.01) and 2.89 times after stimulation with 10 µg/mL Hb-Hp1-1 complexes (*p*-value < 0.01). In HG, the suppression of IL-1β release was not as pronounced as in NG (1 µg/mL: 1.95 times, *p*-value < 0.01; 10 µg/mL: 1.81 times, *p*-value < 0.05). The stimulation with 1 or 10 µg/mL Hb-Hp2-2 complexes did not elicit any significant effect on IL-1β release compared to non-stimulated M(IFNγ), neither in normoglycemic nor in hyperglycemic conditions after 6 h. After 24 h, however, IL-1β secretion was enhanced compared to 6 h after stimulation with 10 µg/mL Hb-Hp2-2 complexes (NG: 2.32 times, *p*-value < 0.01; HG: 1.44 times, *p*-value < 0.01).

The release of the proinflammatory cytokine IL-6 was significantly enhanced in NG 6 h after the stimulation with Hb-Hp1-1 complexes (1 µg/mL: 3.03 times, *p*-value < 0.01; 10 µg/mL: 2.96 times, *p*-value < 0.05 times, Figure 3D). Then, 24 h after the stimulation with Hb-Hp1-1 complexes, suppression of IL-6 secretion was measured in both normo- and hyperglycemia, with the strongest suppression detected in NG after stimulation with 1 µg/mL Hb-Hp1-1 complexes (5.78 times, *p*-value < 0.001) and 3.9 times suppression after stimulation with 10 µg/mL Hb-Hp1-1 complexes (*p*-value < 0.01). In HG, the suppression of IL-6 release was not as pronounced as in NG (1 µg/mL: 3.05 times, *p*-value < 0.001; 10 µg/mL: 2.17 times, *p*-value < 0.05). The stimulation with 1 or 10 µg/mL Hb-Hp2-2 complexes did not elicit any statistically significant effect on IL-6 release after 6h compared to non-stimulated M(IFNγ), neither in normoglycemic nor in hyperglycemic conditions. However, after 24 h, enhanced IL-6 secretion was detected after stimulation with 10 µg/mL Hb-Hp2-2 complexes in NG (3.06 times, *p*-value < 0.01). In hyperglycemic conditions in general, the release of IL-6 was elevated; however, a statistically significant effect could only be detected 24 h after stimulation with 10 µg/mL Hb-Hp2-2 complexes (1.26 times enhancement, *p*-value < 0.05). 

The release of IL-8 was significantly enhanced 6 h after the stimulation with 1 µg/mL Hb-Hp1-1 complexes in NG (2.51 times, *p*-value < 0.05, Figure 3E). Then, 24 h after the stimulation with Hb-Hp1-1 complexes, suppression of IL-8 secretion was detected in both NG and HG, with the strongest suppression detected in NG after stimulation with 1 µg/mL Hb-Hp1-1 complexes (3.06 times, p-value < 0.05) and 2.4 times suppression after stimulation with 10 µg/mL Hb-Hp1-1 complexes (*p*-value < 0.05). In HG, only the stimulation with 10 µg/mL Hb-Hp1-1 complexes suppressed IL-8 release in a statistically significant way (1.67 times, *p*-value < 0.05). The stimulation with Hb-Hp2-2 complexes did not elicit any statistically significant change in IL-8 release after 6 h. Moreover, 24 h after stimulation with 10 µg/mL Hb-Hp2-2 complexes in HG, significantly enhanced IL-8 secretion was detected compared to 6 h (1.48 times, *p*-value < 0.05). In hyperglycemic conditions, a trend towards increased IL-8 secretion was detected 6 and 24 h after Hb-Hp complex stimulation; however, no statistically significant effect was identified due to donor-specific responses.

The secretion of the anti-inflammatory cytokine IL-1RA was enhanced 6 h after stimulation with Hb-Hp1-1 complexes in both normo- and hyperglycemia; however, the effect was only statistically significant in NG (1 µg/mL: 1.8 times, *p*-value < 0.05; 10 µg/mL: 1.66 times, *p*-value < 0.05, Figure 3F). Moreover, 24 h after the stimulation with Hb-Hp1-1 complexes, suppression of IL-1RA secretion was measured in both NG and HG, with the strongest suppression detected in NG after stimulation with 1 µg/mL Hb-Hp1-1 complexes (3.24 times, *p*-value < 0.05) and 1.62 times after stimulation with 10 µg/mL Hb-Hp1-1 complexes (*p*-value < 0.01). Hyperglycemia enhanced IL-1RA secretion after all stimulations; however, the effect was only statistically significant 6 h after stimulation with Hb-Hp2-2 complexes (1 µg/mL: 2.24 times, *p*-value < 0.05; 10 µg/mL: 2.03 times, *p*-value < 0.01) and 24 h after stimulation with 10 µg/mL Hb-Hp1-1 complexes (2.04 times, *p*-value < 0.05). 

Hb-Hp1-1 complex uptake was the strongest stimulus for M(IFNγ) for the acute (6 h) release of proinflammatory cytokines TNFα, IL-1β, IL-6, and IL-8 and anti-inflammatory IL-1RA, where the strongest effect was identified for IL-6 (3.03 times, *p*-value < 0.01). However, 24 h after Hb-Hp1-1 complex uptake (1 µg/mL), the initially detected cytokine release was suppressed in normoglycemic conditions, with the strongest effect being detected for IL-6 (5.78 times; *p*-value < 0.001, Figure 3D) and IL-1β (4.21 times; *p*-value < 0.01, Figure 3C). Hyperglycemia interfered with Hb-Hp1-1-mediated suppression for all cytokines (e.g., IL-6: only 3.05 times suppression in HG, Figure 3D; IL-1β: only 1.95 times suppression in HG, Figure 3C). Contrarily, Hb-Hp2-2 complex uptake did not affect cytokine release in a significant way after 6 h but increased the secretion of all read-out cytokines after 24 h, with the release of IL-6 being stimulated the most (3.06 times, *p*-value < 0.01). Overall, the strongest enhancing effect of hyperglycemia was detected for IL-1β release 24 h after M(IFNγ) was stimulated with 1 µg/mL Hb-Hp1-1 complexes (6.75 times).

## 3. Discussion

In order to sustainably treat the skyrocketing number of patients affected by microvascular complications of diabetes mellitus, it is crucial to broaden our understanding of the immunological mechanisms leading to a derogated control of vascular damage [5,42]. In our study, we addressed the hypothesis that hyperglycemia can convert the silent clearance function of monocytes to an inflammatory response and examined this hypothesis by analysis of the clearance of hemoglobin-haptoglobin complexes. As a result, our study identifies a new mechanism of hyperglycemia-amplified inflammation by affecting hemoglobin-haptoglobin interactions with the innate immune system. In this study, we show for the first time that hyperglycemia interferes with the tolerogenic hemoglobin-haptoglobin scavenging process via CD163 by human primary macrophages, and elevates the production of inflammatory cytokines in response to hemoglobin-haptoglobin complex uptake.

So far, a number of studies have tried to elucidate the role and regulation of CD163 in pathological conditions, such as diabetes mellitus or inflammation, altogether [32,33,43,44]. In recent studies, mainly samples of already differentiated tissue macrophages [33,45], undifferentiated peripheral blood mononuclear cells [32], or the plasma concentration of the shed receptor, soluble CD163 [46,47], were used. Compared to them, our group used human primary peripheral blood macrophages derived from circulating monocytes.

We showed that IFNγ alone is an effective suppressor of CD163 expression on human primary macrophages, thus impairing the scavenging capacity of proinflammatory macrophages. These results, although being observed after a longer duration of cultivation (6 days), correlate with the findings of other studies in which CD163 mRNA and surface expression were decreased on freshly isolated or one-day old peripheral blood monocytes from healthy individuals after IFNγ stimulation [22,26].

Remarkably, we found a discrepancy between CD163 gene and surface expression in M(IL-4) compared to M0. Whereas the mRNA expression of *CD163* was significantly downregulated in M(IL-4) compared to M0, the surface expression of CD163 was not affected by stimulation with IL-4. In agreement with this observation, Staples et al. described a downregulation of *CD163* mRNA expression upon stimulation with IL-4 [48] and both Sulahian et al. and van den Heuvel et al. demonstrated that stimulation with IL-4 did not alter CD163 surface expression compared to M0 [23,27]. Thus, healing macrophages should possess a compensatory mechanism to ensure sufficient levels of surface CD163 to ensure control over the inflammatory response.

Apart from IFNγ- and IL-4-mediated regulation of CD163, hyperglycemia elicited an additional suppression of *CD163* mRNA in M(IFNγ) compared to normoglycemia. This finding is in line with the observation that *CD163* mRNA expression in PBMCs of diabetic individuals was significantly lower compared to PBMCs of healthy subjects [32]. Additionally, clinical studies not only found that pre-diabetic subjects displayed a significant increase in proinflammatory M(IFNγ), but also showed that diabetic patients had an elevated M1/M2 ratio correlating with a higher prevalence of microangiopathy [42,49]. 

The decrease of CD163 surface expression in hyperglycemia is congruent with the results of Levy et al., who found that PBMCs acquired from diabetic individuals expressed significantly less CD163 on their cell surface than those from healthy donors [33]. This reduced scavenging capacity might lead to an elevated heme toxicity contributing to endothelial damage and indicating the susceptibility of the diabetic patient to vascular complications due to dysfunctional control of tissue damage [5,50]. The clinically observed heterogeneity of manifestation and onset of vascular diabetic complications correlates with the observed donor-dependent response of macrophages to hyperglycemia. We show that cytokines, such as IFNγ and IL-4, define the direction of macrophage response, whereas hyperglycemia interferes by enhancing or annulating this cytokine effect.

Suppression of CD163 expression in M(IFNγ) in hyperglycemia raised the question of whether high-glucose conditions additionally have a direct impact on the scavenging function of CD163. Although the two tested variants of CD163′s ligand haptoglobin Hp1-1 and Hp2-2 differ considerably in their molecular structure [34,51], the uptake patterns of Hb-Hp complexes matched CD163 mRNA and surface expression patterns. Remarkably, higher uptake was found for Hb-Hp2-2 complexes compared to Hb-Hp1-1 complexes in all three macrophage subpopulations, correlating with a higher affinity of the Hp2-2 variant for the CD163-binding site located in the scavenger receptor cysteine-rich (SRCR) domain 3 [21,52]. Whether the structure of haptoglobin itself is the crucial factor in the process of Hb-Hp complex internalization is still controversial. An in vitro study performed on monocytes showed that the uptake of Hb-Hp complexes is competitively inhibited by free hemoglobin, thus indicating a common binding site of free and complexed hemoglobin and demonstrating that CD163–hemoglobin interactions are not affected by changes in structure resulting from the binding process of hemoglobin to haptoglobin [53]. This seems to be in contradiction to various other studies describing CD163 as the specific receptor for hemoglobin complexed to haptoglobin but not the free hemoglobin molecule [21,39].

To the best of our knowledge, there have not been any studies reporting a qualitative derogation of the CD163 scavenging function. However, the clinically observed proneness to vascular complications in diabetic patients is enhanced by the limited availability—and therefore limited capacity to mitigate vessel damage—of CD163 in proinflammatory conditions [50].

As no functional impairment of Hb-Hp complex uptake via CD163 was detected, we analyzed whether the uptake itself can provoke an inflammatory activation of macrophages. M(IFNγ) was chosen as the read-out macrophage subpopulation, taking into account that hyperglycemia is able to polarize macrophages towards an M1-like phenotype [13] and the clinical observation of an increased M1/M2 ratio in diabetics [42]. In vitro studies showed the activation of protein-kinase *C*- and casein-kinase-dependent macrophage pathways by cross-linking of cell surface CD163, triggering the release of proinflammatory cytokines, such as IL-1β and IL-6 [23,54,55]. Moreover, it was found in human atherosclerotic plaques that the exposure to Hb-Hp complexes leads to a particular macrophage phenotype, named M(Hb) or Mhem [56,57]. This phenotype is characterized by an abundant expression of surface CD163, downregulated cytokine production, and the lack of lipid withholding [58,59,60]. As these macrophages are particularly present in areas of hemorrhage and neoangiogenesis, a role in plaque vascularization, microvessel leakage, and inflammation of the surrounding endothelium has been suggested [56], thus questioning the long-established notion that CD163^+^ macrophages are involved in the resolution of inflammation [26,50]. Adding to a possible role of CD163 in proinflammatory macrophage activation, alveolar spaces of severely infected COVID-19 lungs contained a large amount of CD163^+^ macrophages as a sign of altered airway macrophage populations and correlating with diffuse alveolar damage and worse patient outcomes [61]. Moreover, the serum levels of soluble CD163, as a marker of macrophage activation, were enhanced in COVID-19 patients [62,63]. 

As a possible factor contributing to diverging results, the haptoglobin variants Hp1-1 and Hp2-2 were found to have not only differences in function, but also in the involvement in pathological conditions. For instance, the clearance and antioxidant capacity of Hp1-1 by binding hemoglobin was superior to the clearance of Hp2-2 [38,39]. The release of anti-inflammatory IL-10 in response to the binding of Hb-Hp1-1 complexes to CD163 was increased compared to Hb-Hp2-2 complexes [64]. Regarding the clinical significance of the different haptoglobin variants, it has been shown in longitudinal prospective studies that the Hp2-2 phenotype is an independent risk factor for the development of cardiovascular disease in diabetic individuals in comparison with the homozygous Hp1 variant [34,40]. Additionally, it has been demonstrated that CD163 is downregulated in macrophages of atherosclerotic plaques of diabetic patients with the Hp2-2 genotype, indicating a constrained hemoglobin clearing capacity [33]. 

To detect the inflammatory response of Hb-Hp scavenging in hyperglycemic conditions, we selected a number of read-out cytokines displaying the complex interaction and different stages of an inflammatory reaction. In healthy individuals, the process of inflammation serves a homeostatic purpose, containing pro- and anti-inflammatory phases [65]. In individuals suffering from type 2 diabetes mellitus, however, the balance is tilted towards the proinflammatory side, manifested by an upregulation of proinflammatory intracellular pathways [66] and an elevation in circulating inflammatory markers, such as C-reactive protein, IL-6, and TNFα [67,68,69].

We showed that hyperglycemia enhanced the proinflammatory response of M(IFNγ) to Hb-Hp complex uptake by stimulating the production of TNFα, IL-1β, IL-6, IL-8, and IL-1RA, supporting the observation that hyperglycemia itself can induce the secretion of proinflammatory cytokines [6].

A statistically significant increase in TNFα secretion, the ‘classical player’ of the acute-phase immune response [70], could only be detected 6 h after the stimulation with Hb-Hp complexes in hyperglycemia, regardless of the present haptoglobin variant. This finding corresponded to the clinical observation of elevated TNFα-levels being detected in newly diagnosed diabetic patients compared to a healthy cohort [71,72]. 

We found that in response to the uptake of Hb-Hp1-1 complexes, out of all the tested cytokines, the most pronounced effects were detected for IL-6. IL-6 plays a key role in the regulation of acute-phase protein production by hepatocytes, upregulating, among others, the secretion of haptoglobin and C-reactive protein [73]. Elevated IL-6 release might contribute to diabetes progression, as an in vitro study reported the induction of cellular insulin resistance of hepatocytes by IL-6-triggered impairment of insulin receptor signal transduction [74]. Another in vitro study showed that lower concentrations (20 µg/mL) of Hb-Hp1-1 complexes induced the secretion of proinflammatory IL-6 while higher concentrations (100 µg/mL) of Hb-Hp1-1 complexes, however, led to increased CD163 surface expression on monocytes, in terms of a positive anti-inflammatory feedback loop [75].

We found that hyperglycemia increased the secretion of both IL-1β and its natural inhibitor IL-1RA, confirming our previous data [6]. Increased IL-1RA secretion could be a possible compensation mechanism or negative feedback for the enhanced release of IL-1β, whose serum level was found to be elevated in diabetic patients, contributing to insulin resistance and progression of atherosclerotic lesions in obesity [76]. A compensatory role of IL-1RA might also be an explanation for the controversial observations concerning IL-1RA levels in hyperglycemia: on the one hand, elevated IL-1RA levels could be correlated with insulin resistance [75,77]; on the other hand, IL-1RA was found to diminish markers of inflammation in the blood samples of diabetic patients and enhance beta cell function [78].

In general, Hb-Hp1-1 complex uptake was the strongest stimulus for M(IFNγ) for acute (6 h) cytokine release; however, cytokine secretion was diminished after 24 h. Contrarily, Hb-Hp2-2 complex uptake resulted in the increased secretion of all read-out cytokines after 24 h, indicating a transcriptional upregulation of cytokine production. Our in vitro finding that Hb-Hp2-2 complex uptake is able to create a long-lasting elevated release of proinflammatory cytokines is in line with the numerous clinical observations of Hp2-2 possessing a lesser antioxidative capacity than Hp1-1 [38,39], inducing a lesser anti-inflammatory response than Hp1-1 [64] and even being an independent risk factor for the development of cardiovascular disease in diabetic patients [34,40]. 

As an element of the pathophysiology of diabetes, these mechanisms promote the low-grade chronic inflammation present in the vascular system of diabetic patients and support the development of vascular complications. This shows that—in addition to a more proinflammatory setting—diabetes mellitus is a disease characterized by less effective anti-inflammatory damage control [5,65]. 

However, our study was limited to an ex vivo analysis of the response of primary human monocyte-derived macrophages, and the examination of metabolic syndrome patients or patients with untreated diabetes is necessary to validate the effect of hyperglycemia on the clearance of Hp-Hb complexes. The model we used enabled specific identification of the effect of hyperglycemia on Hp-Hb clearance and inflammatory programming in human monocyte-derived macrophages. Further research using monocytes isolated from patients with non-compensated hyperglycemia is needed to validate our findings, and to determine the level of contribution of the Hp-Hb-induced inflammatory monocyte response to systemic inflammation. We also believe that in the future, the effect of currently used medications for diabetic patients should also be analyzed for their ability to minimize Hp-Hb-induced inflammation.

## 4. Materials and Methods

### 4.1. Isolation of Monocytes and Cultivation of Macrophages

Human monocytes were isolated by density gradient centrifugation from buffy coats of individual healthy donors provided by the blood bank of the DRK-Blutspendedienst Baden-Württemberg-Hessen in Mannheim [79]. In total, 30 mL of buffy coat were mixed with Dulbecco’s Phosphate-Buffered Saline (DPBS, Biochrom, Berlin, Germany) at a 1:1 ratio. The mixture was added on top of 15 mL of Biocoll (Biochrom, Berlin, Germany) and centrifuged for 30 min at 420 rcf (Hettich, Rotina, Tuttlingen, Germany). From the white interphase of the Ficoll gradient, peripheral blood mononuclear cells (PBMCs) were collected and washed twice with DPBS. The Percoll gradient was prepared using 13.5 mL of Percoll^TM^ (GE Healthcare, Solingen, Germany), 15 mL of Minimum Essential Medium (MEM, Sigma Aldrich, St. Louis, MO, USA), and 1.5 mL of Earle’s Balanced Salt Solution (EBSS, Sigma Aldrich, St. Louis, MO, USA). PBMCs were layered on the Percoll gradient and the coating containing monocytes was collected after 30 min of centrifugation at 420 rcf (Hettich, Rotina, Tuttlingen, Germany). After two washing steps, the cells were mixed with CD14^+^ magnetic beads according to the cell sorting protocol of Miltenyi Biotech’s monocyte isolation kit (Bergisch Gladbach, Germany). The monocytes were counted using a Casy^TM^ cell counter (OLS OMNI Life Science GmbH & Co. KG, Bremen, Germany) and the purity was controlled by flow cytometry. Ex vivo differentiation of monocyte-derived macrophages in normoglycemic and hyperglycemic conditions was performed as previously described [6,7]. Briefly, 1 × 10^6^ monocytes per ml were cultured in Serum-Free Medium (Life Technology, Merck KGaA, Darmstadt, Germany) containing 5 mM glucose or 25 mM glucose for a duration of 6 days at 37 °C in 7.5% CO_2_. The added cytokines were purchased from TEBU Preprotech (Frankfurt am Main, Germany). Human IFNγ was used at a concentration of 100 ng/mL, human IL-4 at a concentration of 10 ng/mL, dexamethasone at a concentration of 10^−8^ M, and human M-CSF at a concentration of 5 ng/mL. Cell viability was assessed using alamarBlue^TM^ (Supplementary Figure S1).

### 4.2. RNA Samples, cDNA Synthesis, and Real-Time PCR Analysis

For RNA isolation, the cell lysis was performed directly in plastic Petri dishes. The RNA was isolated using an E.Z.N.A. total RNA kit (Omega Bio-Tek Inc., Norcross, GA, USA) according to the manufacturer’s protocol. Up to 1 μg of RNA was added to 1 μg of DNase I (RNase free, Thermo Fisher Scientific, Dreieich, Germany). To synthesize cDNA, Oligo DT primer and Reverse Transcriptase purchased from Thermo Fisher Scientific, Germany were used (RevertAid RT Transcription kit). The cDNA was diluted with ddH_2_O at a 1:10 ratio. 

Real-time PCR analysis of *CD163* was performed using 0.5 µL of cDNA. The mRNA level was quantified using TaqMan PCR mastermix (Applied Biosystems, Darmstadt, Germany). As a reference gene, 18s rRNA was used to normalize the expression levels of the genes analyzed. The primers for human *CD163* were designed with MWG Biotech/GeneScript. As a forward primer F2101 (TAGTGAGTGTGGGCACAAGG), as a reversed primer R2102 (CCGACTGCAATAAAGGATGA), and as probe Pr2102 (CACAACAGGTCGCTCATCCCG), all purchased from Eurofins Genomics, Ebersberg, Germany, were found to have an appropriate efficacy. The amplification was performed by a Light Cycler 480 system (Roche Life Science, Penzberg, Germany). The values are displayed as the average of the measurement of three replicates.

### 4.3. Flow Cytometry 

Flow cytometry analysis of CD163 surface expression on differentially activated macrophages after 6 days of culture in low (5mM) and high (25 mM) glucose conditions was performed at the Flow Cytometry Core Facility of the Institute of Transfusion Medicine and Immunology, Medical Faculty Mannheim, University of Heidelberg, Mannheim, Germany. The cells were stained with anti-human CD163 and anti-CD14 for 30 min on ice using the following antibodies: FITC-conjugated mouse IgG1κ anti-CD14 antibody, APC-conjugated mouse IgG1κ anti-human CD163, FITC-conjugated mouse IgG1κ isotype control; APC-conjugated mouse IgG1κ isotype control (all purchased from eBioscience Inc., San Diego, CA, USA). Human TruStain FcX (Fc Receptor blocking solution) was purchased from Biolegend Inc., San Diego, CA, USA. Fluorescence intensity was measured using a BD FACS Canto II (Becton Dickinson, Franklin Lakes, NJ, USA) equipped with the BD FACSDiva software (Becton Dickinson, Franklin Lakes, NJ, USA). The values are displayed as the average of the measurement of three replicates. Data analysis was performed using the FlowJo software (FlowJo, Becton Dickinson, Franklin Lakes, NJ, USA). The gating of monocytes and macrophages was based on forward-sideward scatter profiles (shown in Supplementary Figure S2). The cells were stained with a PE-conjugated mouse IgG2aκ anti-human HLA-DR antibody (Biozol Diagnostica Vertrieb GmbH, Eching, Germany), which was used as a cell viability marker (Supplementary Figure S3).

### 4.4. ELISA

Supernatants of macrophages were harvested on day 6 of cultivation in NG and HG conditions. The concentrations of the cytokines IL-1β, IL-1RA, IL-6, IL-8, and TNFα in the supernatants were measured by ELISA (Human IL-1β DuoSet ELISA kit (dilution of samples: 1:2), Human IL-1RA DuoSet ELISA kit (dilution of samples: 1:50), Human IL-6 DuoSet ELISA kit (dilution of samples: 1:2), Human IL-8 DuoSet ELISA kit (dilution of samples: 1:50), and Human TNFα DuoSet ELISA kit (dilution of samples: 1:2)) according to the protocols of the manufacturer (R&D Systems, Minneapolis, MN, USA). After bringing all components to room temperature, the plates were prepared by diluting the capture antibody to a working concentration in PBS. After incubation overnight, the plate was washed (0.05% Tween 20 in PBS; pH: 7.2–7.4) and blocked by Reagent Diluent (1% BSA in PBS; pH: 7.2–7.4). The standard was diluted according to the manufacturer’s protocol. Samples were thawed and diluted as indicated above. The standard was pipetted in a serial dilution in the first two columns of the plate. After adding the samples to the rest of the plate, a 2-h incubation, and consecutive washing step, 100 µL of detection antibody were added per well. After another incubation period of 2 h and washing step, 100 µL of Streptavidin-HRP were added to each well. After a 20-min period of incubation in the dark, 100 µL of substrate solution (1:1 mixture of Color Reagent A (H_2_O_2_) and Color Reagent B (Tetramethylbenzidine)) were added, followed by 20 min of incubation. In total, 50 µL of stop solution (2N H_2_SO_4_) were added. Immediately after adding the stop solution, the optical density of each well was determined. A TECAN Infinite 200 microplate reader (Thermo Fisher Scientific, Dreieich, Germany) set to 450 nm was used. The reference wavelength was set to 570 nm. All samples were measured in duplicate.

### 4.5. Endocytosis of Hp-Hb Complexes

Human haptoglobin variants Hp1-1 and Hp2-2 (Sigma Aldrich, St. Louis, MO, USA) were fluorescently labeled with Alexa Fluor^®^ 488 according to the Alexa Fluor^®^ 488 Protein Labeling Kit procedure (Molecular Probes, Eugene, OR, USA). The extent of labeling was determined using the Thermo Scientific^TM^ Pierce^TM^ BCA Protein Assay Kit (Pierce Biotechnology, Waltham, MA, USA). To build hemoglobin-haptoglobin complexes, human hemoglobin (A_0_, 1 mg/mL, Sigma Aldrich, St. Louis, MO, USA) was added to previously labeled Hp1-1 or Hp2-2 and incubated at 37 °C for 10 min on a rotator. Subsequently, the complexes were added to macrophages cultured for 6 days in NG and HG conditions. Alexa Fluor^®^ 488-labeled acLDL (Life Technology, Merck KGaA, Darmstadt, Germany) was used as a positive control for macrophage endocytic activity. Macrophages were incubated with fluorescently labeled ligands (10 µg/mL of Hb-Hp1-1 complexes, 10 µg/mL of Hb-Hp2-2 complexes, or 5 µg/mL Alexa Fluor^®^ 488-labeled acLDL) for 30 min at 37 °C and 7% CO_2_. After the endocytosis procedure, macrophages were placed on ice for 60 min. Harvested cells were washed with PBS, and the fluorescence intensity was measured using BD FACS Canto II (Becton Dickinson, Franklin Lakes, NJ, USA) equipped with BD FACSDiva software (Becton Dickinson, Franklin Lakes, NJ, USA). The MFI values of Hb-Hp complex uptake were measured in triplicates and calculated by subtracting the MFI values of ‘no ligand’ controls.

### 4.6. Immunofluorescent Staining and Confocal Microscopy

First, 1 × 10^6^ monocytes per ml were cultured in Serum-Free Medium (Life Technology, Merck KGaA, Darmstadt, Germany) containing 5 or 25 mM glucose for 6 days. The following cytokines were added: human IFNγ (100 ng/mL) or human IL-4 (10 ng/mL); dexamethasone (10^−8^ M); and human M-CSF (5 ng/mL) (all purchased from TEBU Preprotech (Frankfurt am Main, Germany)). Human haptoglobin variants Hp1-1 and Hp2-2 (Sigma Aldrich, St. Louis, MO, USA) were fluorescently labeled with Alexa Fluor^®^ 488 according to the Alexa Fluor^®^ 488 Protein Labeling Kit procedure (Molecular Probes, Eugene, OR, USA). To build hemoglobin-haptoglobin complexes, human hemoglobin (A_0_, 1 mg/mL, Sigma Aldrich, St. Louis, MO, USA) was added to previously labeled Hp1-1 or Hp2-2 and incubated at 37 °C for 10 min on a rotator. Subsequently, the complexes were added to the macrophages after 6 days of culture. Alexa Fluor^®^ 488-labeled acLDL (Life Technology, Merck KGaA, Darmstadt, Germany) was used as a positive control for macrophage endocytic activity. The cells were incubated with fluorescently labeled ligands for 30 min at 37 °C under 7% CO_2_. Then, the macrophages were harvested and transferred onto glass slides using cytospin centrifuge (Thermo Fisher Scientific, Dreieich, Germany). Fixation of cells on glass slides by PFA and staining was performed as previously described [79]. As primary antibody goat polyclonal anti-human CD163 and as isotype control goat IgG ‘normal goat IgG’ (both purchased from Santa Cruz, Biotechnology, Dallas, TX, USA) were used. As secondary antibody donkey anti-goat Cy3 (Dianova, Hamburg, Germany) was used. Nuclei were visualized by DRAQ5 (New England BioLabs, Ipswich, MA, USA).

Confocal laser scanning microscopy was performed using a Leica TCS SP2 laser scanning spectral confocal microscope equipped with a 63 × 1.32 objective (Leica Microsystems, Wetzlar, Germany). An argon laser with an emission wavelength of 488 nm, a krypton laser with an emission wavelength of 568 nm, and a helium/neon laser with an emission wavelength of 633 nm were used to perform excitation. Data were acquired and analyzed with the Leica Confocal software (Leica Microsystems, Wetzlar, Germany). Two- and three-color images were acquired using the sequential scan mode.

### 4.7. Inflammatory Response Assay

CD14^+^ monocytes of 5 healthy donors were differentiated for 6 days in a serum-free medium complemented with human IFNγ (100 ng/mL) in normal (5 mM) and high (25 mM) glucose conditions. After 6 days of culturing, the macrophages were stimulated with hemoglobin-haptoglobin1-1 complexes or hemoglobin-haptoglobin2-2 complexes in 2 different concentrations (1 and 10 µg/mL). Supernatants were obtained 6 and 24 h after stimulation with the complexes. Concentrations of IL-1β, IL-1RA, IL-6, IL-8, and TNFα were measured by ELISA according to the protocols of the manufacturer (R&D Systems, Minneapolis, MN, USA) and measured in duplicates. A TECAN Infinite 200 microplate reader (Thermo Fisher Scientific, Dreieich, Germany) set to 450 nm was used to read the plates. The reference wavelength was set to 570 nm; again, all samples were measured in duplicate.

### 4.8. Statistical Analysis

Statistical analysis was performed using GraphPad Prism 8 software (GraphPad Software Inc., San Diego, CA, USA). The significance of the difference between the two groups of experimental data was determined using the Wilcoxon matched-pairs rank test or a paired *t* test. We considered a two-tailed *p*-value of less than 0.05 to indicate statistical significance (confidence level 95%). All graphs were created using the software GraphPad Prism 8.

## 5. Conclusions

Here, we show for the first time that hyperglycemia can affect essential monocyte/macrophage clearance functions required to eliminate Hb-Hp complexes. Hyperglycemia has already been identified as a direct driver of inflammatory cytokine release in monocytes and macrophages. We found that hyperglycemia does not affect internalization of Hp1-1 and Hp2-2 genetic variants, neither in proinflammatory nor in anti-inflammatory macrophages. However, we identified that hyperglycemia enhances the release of inflammatory cytokines in response to scavenging of Hb-Hp complexes via CD163 in M(IFNγ), converting the silent Hb-Hp complex clearing process needed to prevent vascular damage into an inflammatory process. These mechanisms can promote low-grade chronic inflammation in individuals with metabolic syndrome and non-treated diabetes, and can predispose these patients to the development of micro- and macrovascular complications.

## Supplementary Materials

Supplementary materials can be found at https://www.mdpi.com/article/10.3390/ijms23031385/s1.

## Abbreviations

CD	cluster of differentiation
DCCT	Diabetes Control and Complications Trial
HG	high glucose conditions, hyperglycemia (25mM)
Hb	hemoglobin
Hp	haptoglobin
M0, M(Control)	non-stimulated macrophages
M(IFNγ)	macrophages stimulated with IFNγ
M(IL-4)	macrophages stimulated with IL-4
M1	classically activated macrophages
M2	alternatively activated macrophages
MFI	mean fluorescence intensity
NG	normal glucose conditions, normoglycemia (5mM)
no compl	‘no complexes’
PBMC	peripheral blood mononuclear cells
PFA	paraformaldehyde
RAGE	receptor for advanced glycation endproducts
UKPDS	United Kingdom Prospective Diabetes Study
