# Supplementary material for: Hyperglycemia Induces Inflammatory Response of Human Macrophages to CD163-Mediated Scavenging of Hemoglobin-Haptoglobin Complexes
Source: Int J Mol Sci. 2022 Jan 26;23(3):1385. doi: 10.3390/ijms23031385 (PMC8836198; doi:10.3390/ijms23031385)
Supplement: Supplementary file 1 [file ijms-23-01385-s001.zip › ijms-1558499-supplementary.pdf]

International Journal of
Molecular Sciences

Supplementary Materials

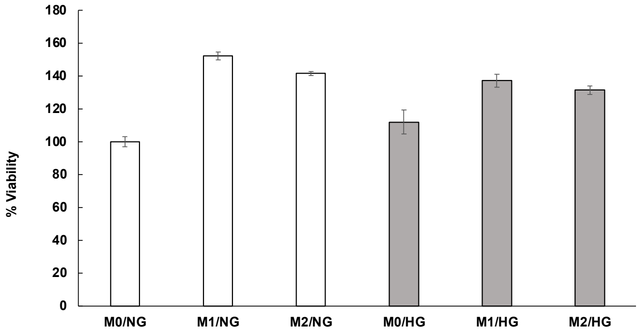

Figure S1. Cell viability assessment of non-stimulated (M0), IFNγ-stimulated (M1) and IL-4-stimulated (M2) macrophages using alamarBlue™. Human monocyte-derived macrophages were stimulated with IFNγ or IL-4 or left without cytokine stimulation and cultured for 6 days in normal (5 mM, NG) and high (25 mM, HG) glucose conditions. On day 6, fluorescence intensity was measured after 3 h of incubation with alamarBlue™. Each bar represents the mean percentage of viability of 4 donors with SEM; all bars were normalized to M0/NS. For statistical analysis, a paired t test was performed.

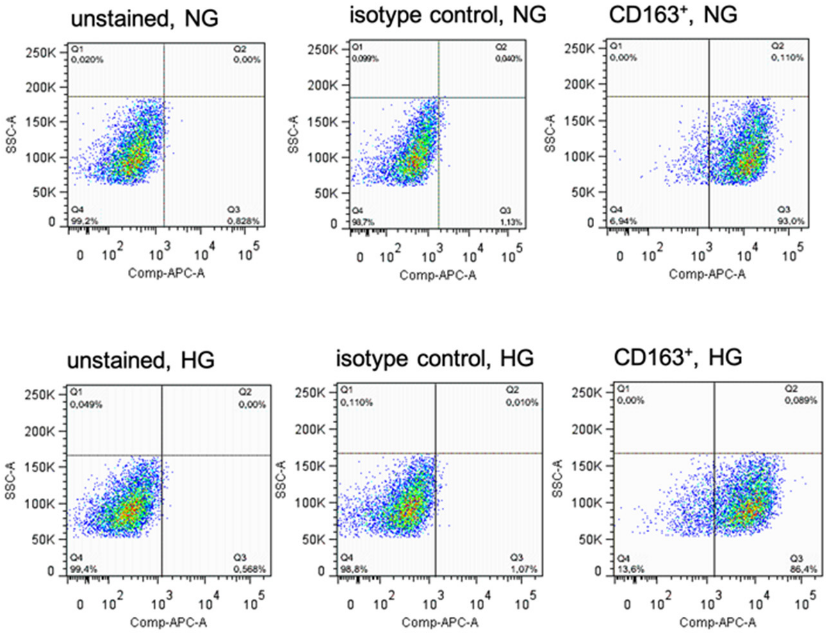

Figure S2. Gating process for CD163+ M(IFNγ). Exemplary depiction of gating process for CD163+ IFNγ-stimulated monocyte-derived macrophages after being cultured for 6 days in normal glucose (5 mM, NG) and high glucose (25 mM, HG) conditions. Graphs depict individual forward-sideward scatter profiles for one donor.

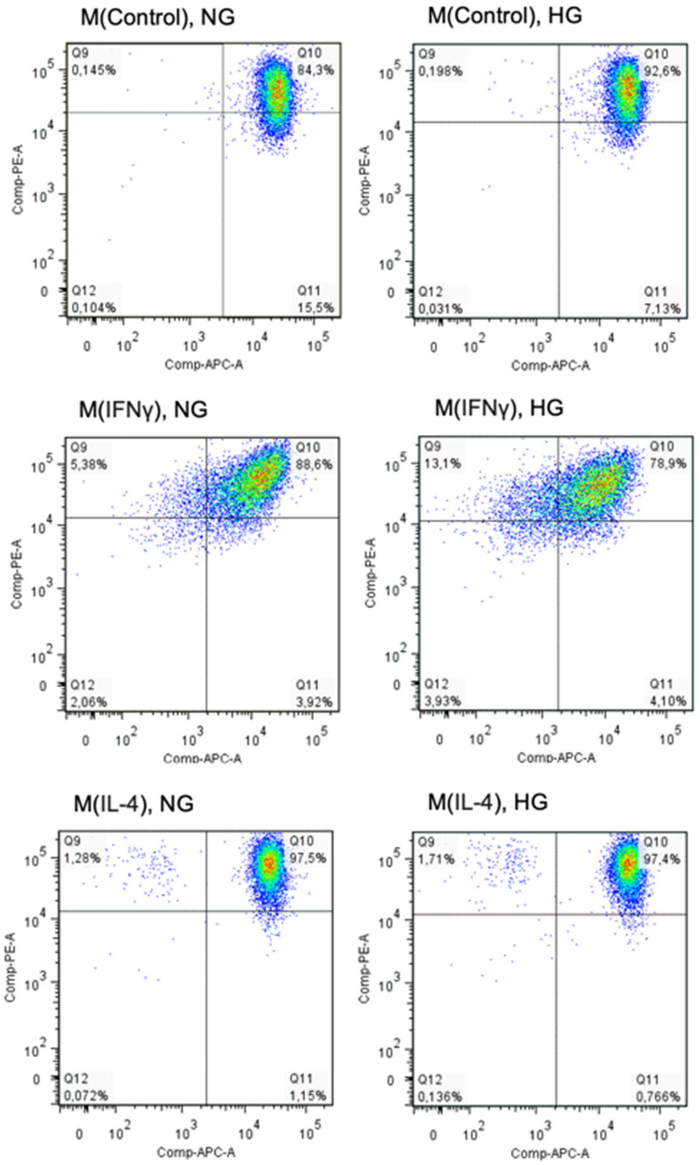

Figure S3. Flow cytometry analysis of CD163^{+}HLA-DR^{+} monocyte-derived macrophages. Exemplary flow cytometry analysis of CD163^{+}HLA-DR^{+} monocyte-derived macrophages. Monocyte-derived macrophages were cultured for 6 days in normal glucose (5 mM, NG) and high glucose (25 mM, HG) conditions and stimulated with either IFNγ or IL-4 or without further cytokines (M(Control)). Graphs depict individual forward-sideward scatter profiles for one donor. To determine CD163 surface expression, an APC-conjugated mouse IgG1κ anti-human CD163 antibody was used (x-axis), to depict HLA-DR surface expression, a PE-conjugated mouse IgG2aκ anti-human HLA-DR antibody was used (y-axis).

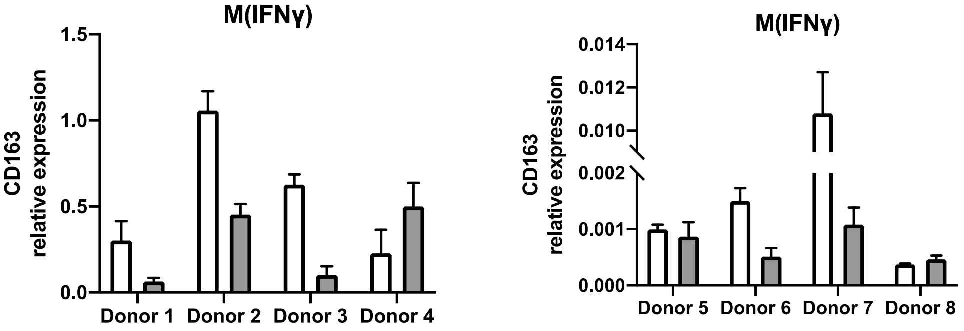

Figure S4. RT-PCR analysis of CD163 gene expression in IFNγ-stimulated macrophages in Individual donor. RT-PCR analysis of CD163 gene expression in IFNγ-stimulated macrophages. Human monocyte-derived macrophages were cultured for 6 days in normal glucose (5 mM, NG, white bars) and high glucose (25 mM, HG, grey bars) conditions. Graphs depict individual values of 8 donors (mean ± SD; each condition was measured three times for every donor).

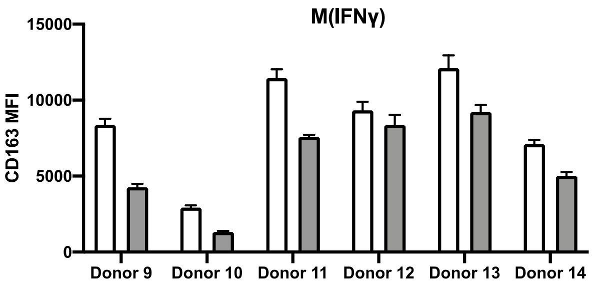

Figure S5. Flow cytometry analysis of CD163 surface expression in IFNγ-stimulated macrophages in individual donors. Flow cytometry analysis of CD163 surface expression in IFNγ-stimulated macrophages. Monocyte-derived macrophages were cultured for 6 days in normal glucose (5 mM, NG, white bars) and high glucose (25 mM, HG, grey bars) conditions. Graph depicts individual values of 6 donors. The values are displayed each as the average of the measurement of three replicates ± SD.
